# Heart rate during hyperphagia differs between two bear species

**DOI:** 10.1098/rsbl.2018.0681

**Published:** 2019-01-16

**Authors:** Boris Fuchs, Koji Yamazaki, Alina L. Evans, Toshio Tsubota, Shinsuke Koike, Tomoko Naganuma, Jon M. Arnemo

**Affiliations:** 1 Department of Forestry and Wildlife Management, Faculty of Applied Ecology and Agricultural Sciences, Inland Norway University of Applied Sciences, Campus Evenstad, 2418 Elverum, Norway; 2 Department of Forest Science, Tokyo University of Agriculture, 1-1-1 Sakuragaoka, Setagaya-Ku, Tokyo, Japan; 3 Department of Environmental Veterinary Sciences, Faculty of Veterinary Medicine, Hokkaido University, Kita18, Nishi9, Kita-Ku, Sapporo, Hokkaido, Japan; 4 Institute of Global Innovation Research, Tokyo University of Agriculture and Technology, 3-5-8 Saiwai, Fuchu-city, Tokyo, Japan; 5 United Graduate School of Agricultural Science, Tokyo University of Agriculture and Technology, 3-5-8 Saiwai, Fuchu-city, Tokyo, Japan; 6 Department of Wildlife, Fish and Environmental Studies, Faculty of Forest Sciences, Swedish University of Agricultural Sciences, 901 83, Umeå, Sweden

**Keywords:** hyperphagia, heart rate, Asian black bear, brown bear, Ursus

## Abstract

Hyperphagia is a critical part of the yearly cycle of bears when they gain fat reserves before entering hibernation. We used heart rate as a proxy to compare the metabolic rate between the Asian black bear (*Ursus thibetanus*) in Japan and the Eurasian brown bear (*Ursus arctos*) in Sweden from summer into hibernation. In the hyperphagic period, black bears feed on fat- and carbohydrate-rich hard masts whereas brown bears feed on sugar-rich berries. Availability of hard masts has quantitative and spatial annual fluctuations, which might require increased activity and result in intraspecific stress. Using generalized additive mixed models we analysed the differences in heart rate between the two species. Black bears had decreased heart rates during summer but had doubled heart rate values throughout the hyperphagic period compared to brown bears. This letter illustrates the different physiological consequences of seasonal differences in food availability in two species of the same genus dealing with the same phenological challenge.

## 1. Background

Seasonal changes in climate and food availability lead to a variety of adaptations across animal species. Migration, hibernation and food caching are important strategies during periods of low food availability and are used in varying degrees and combinations. Asian black bears (*Ursus thibetanus*) and brown bears (*Ursus arctos*) are hibernators and display hyperphagia [1,2]. In Scandinavia and in Japan, bears den for 5 to 6 months depending on weather conditions, food abundance and reproductive status [3,4].

During hyperphagia, berries compose 68% of the brown bears' diet [5] and body mass increases 65% for females [6]. Berries are composed primarily of carbohydrates that are easily converted into fat reserves, and hard masts are not available [5]. The pre-hibernation diet of black bears is primarily (60–86%) composed of hard masts [7], which have high lipid (nuts) or carbohydrate (acorns) content [8].

We sought to compare the metabolic consequences of the two feeding strategies during hyperphagia using heart rate (HR) as a proxy for metabolic rate [9,10] and to discuss the ecological implications.

## 2. Material and methods

Black and brown bears were captured in barrel traps or by helicopter [11,12]. We deployed HR loggers (Milli-HRT, Star-Oddi, Iceland) in three adult female black bears in Ashio-Nikko, Japan and Reveal XT (Medtronic Inc., Minnesota) HR loggers in four female brown bears in Dalarna, Sweden (table 1). In both cases, the loggers were surgically implanted subcutaneously [11]. The loggers were removed (HRT) or downloaded (Reveal XT) approximately 1 year later. Loggers recorded HR in bpm at 10 min (HRT) and 2 min (Reveal XT) intervals.

**Table 1.** Bears (all females) included in the study. Body mass was measured post-hibernation, age either estimated using tooth annual layers (Japan), or known date of birth (Sweden) and den entry day (day of the year) estimated from heart rate.

ID	body mass (kg)	age (years)	winter	species	den entry day
AF 45	38	6	2014/2015	Asian black bear	300
AF 55	44	4	2014/2015	Asian black bear	323
AF 19	44	9	2016/2017	Asian black bear	324
W1304	49	4	2016/2017	Eurasian brown bear	297
W1401	43	3	2016/2017	Eurasian brown bear	301
W1407	48	2	2015/2016	Eurasian brown bear	315
W1408	53	3	2016/2017	Eurasian brown bear	308

All data handling and analysis was done with R v. 3.4.2 [13]. For analysis we calculated the daily mean HR, resulting in 174 to 222 values per individual. Den entry date was determined, using the first day after September 1st with a daily mean HR < 40 bpm [14]. We zeroed the HR data to the day of den entry to compare the change in HR over time across individuals. We then fitted generalized additive mixed models using the function ‘bam’ [15,16]. As response variable, we added daily mean HR. As fixed terms, we fitted a smooth term for pre den entry in days (time), an ordered factor for the species, and a smooth term with species and time, resulting in an interaction-like term. As random structure, we allowed each bear a random intercept and slope. Within this model, we got a fitted nonlinear regression line for each bear and it was possible to determine differences between the groups at any given time. We added an autoregressive model (AR1) structure. The autoregression parameter (*ρ*) was based on the autocorrelation factor of the standardized residuals at lag 1. We considered the HR significantly different between the species on days when the 95% simultaneous confidence intervals of the modelled difference did not overlap with zero. For a more detailed description of the model see the electronic supplementary material.

## 3. Results

**Figure 1.**
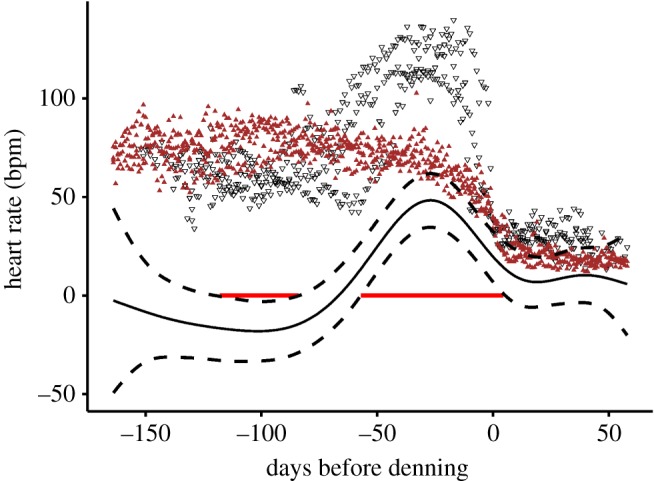
Daily mean heart rate of three Asian black bears (black, open triangles) and four Eurasian brown bears (brown triangles) from June to the end of the year. At time zero bears entered denning period (mean date November 5th). The fitted difference between the species is shown as solid black lines with the 95% c.i. and is considered significant when it did not overlap 0 (red, horizontal solid line). (Online version in colour.)

Black bears had a lower HR compared to the brown bears prior to the hyperphagic period (121 to 84 days prior to hibernation, black bear: 60, ± 8.1 bpm, brown bear: 79, ± 8.6 bpm, mean ± s.d.) (figure 1). During the hyperphagic period, black bears displayed an increase in HR culminating at a mean of 119 ± 9.6 bpm 35 to 20 days prior to denning. The difference between the species was significant from 57 days prior to denning to 5 days post denning (figure 1). At peak, the HR of black bears was about 50 bpm higher than brown bears. After den entry, black bears decreased HR to 30 ± 7 bpm and brown bears to 20 ± 5 bpm but the difference was no longer significant (figure 1). Both species started hibernation around November 1st (mean day of the year 310, ranging from 297 to 324).

## 4. Discussion

Mean daily HR for three female black bears and four female brown bears showed distinct differences both in absolute values as well as over time. We found three different periods. (i) Black bears had a summer dip from July to mid-August, with significantly lower HR than brown bears. (ii) Towards hyperphagia, the HR of black bears nearly doubled within 50 days from 64 bpm during the summer dip to 110 bpm at the beginning of October. (iii) From peak hyperphagia, black bears’ HR dropped to hibernation values below 40 bpm within 25 days. Brown bears decreased HR to hibernation values over a similar amount of time. During early hibernation, the HR of the two species did not differ significantly.

Black bears in Japan display a bimodal activity pattern with peaks in early summer and autumn and decreased activity in late summer, likely owing to low food availability and quality [12]. HR is related to activity and nutritional intake [17] and in the Swedish study area activity does not decrease during summer [4] nor do we have indications of reduced food availability. We hypothesize a resource-induced decrease in metabolism in black bears during summer, potentially paired with species-specific differences explaining the summer dip in HR.

The dramatically higher peak in HR in the black bear during hyperphagia indicates an increase in metabolic rate [10]. The higher metabolic rate could be owing to a variety of factors, including increased activity levels. Black bears in the Japanese study area primarily use Mizunara oak (*Quercus crispula*) and oak production was poor in the studied years. However, Mizurana masting is non-synchronized and rich patches were available also in poor production years [18]. Foraging behaviour might be more energy intense when bears need to search for rich patches and feeding in poor patches will take more time. For example, black bears in this study area climb trees more frequently in years of poor masting [19]. Aggregation of black bears at rich patches could have contributed to intraspecific stress and additionally elevated activity and, thus HR.

Comparing HR during hyperphagia within the black bears but between good and poor mast years might unravel whether increased HR is related to feeding behaviour and activity or to digestion-related factors. Japanese black bears den later in years of rich mast production [7], spending more time and less energy on feeding, conceivably even resulting in similar HR patterns to the brown bears. Lipid metabolism requires more time and energy than glucose metabolism and, if relevant, the HR of black bears would remain elevated in good mast years.

We found highly different physiological consequences in two related species dealing with the same phenological challenge of high seasonal differences in food availability. These differences may change temporally from year to year and spatially with latitude. Bears counter that environmental variation with physiological plasticity. For example, they adapt to the available food resource from being mostly vegetarian to mostly carnivorous with great variation in body size [20], they adjust hibernation phenology to weather conditions, climate and food availability [3,4] or, as indicated in this study, they vary in metabolism depending on food composition. Physiological plasticity might be a major contributor to the bears’ wide distribution and is a promising feature in times of changing climate.

## Supplementary Material

R-code

## Supplementary Material

R-code

## Data accessibility

Data are available from the Dryad Digital Repository [21].
