# Supplementary material for: Heart rate during hyperphagia differs between two bear species
Source: Biol Lett. 2019 Jan 16;15(1):20180681. doi: 10.1098/rsbl.2018.0681 (PMC6371904; doi:10.1098/rsbl.2018.0681)
Supplement: R-code [file rsbl20180681supp2.pdf]

Heart rate during hyperphagia differs between two bear species

Boris Fuchs[1], Koji Yamazaki[2], Alina L. Evans[1], Toshio Tsubota[3], Shinsuke Koike[4],[5], Tomoko Naganuma[5], Jon M. Arnemo[1],[6]

22 November 2018

[1]Department of Forestry and Wildlife Management, Faculty of Applied Ecology and Agricultural Sciences, Inland Norway University of Applied Sciences, Campus Evenstad, NO-2418 Elverum, Norway
[2]Department of Forest Science, Tokyo University of Agriculture, 1-1-1 Sakuragaoka, Setagaya-Ku, Tokyo, Japan
[3]Department of Environmental Veterinary Sciences, Faculty of Veterinary Medicine, Hokkaido University, Kita18, Nishi9, Kita-Ku, Sapporo, Hokkaido, Japan
[4]Institute of Global Innovation Research, Tokyo University of Agriculture and Technology, 3-5-8 Saiwai, Fuchu-city, Tokyo, Japan
[5]United Graduate School of Agricultural Science, Tokyo University of Agriculture and Technology, 3-5-8 Saiwai, Fuchu-city, Tokyo, Japan
[6]Department of Wildlife, Fish and Environmental Studies, Faculty of Forest Sciences, Swedish University of Agricultural Sciences, SE-901 83, Umeå, Sweden

Data description

```
hr <- read.table(file="DataHyperphagia.txt", sep=";",header=T,as.is=T)
```

Mean daily heart rate (HR) from three female, free-ranging Asiatic black bears *(Ursus tibetanus)* in Japan and four female, free-ranging brown bears *(Ursus arctos)* in Sweden. We focused on the hyperphagic period prior to hibernation.

Research question: When and to what extent does HR differ between the two species?

For this analysis we followed the tutorial from Jacolien Van Rij "Overview GAMM analysis of time series data". http://jacolienvanrij.com/Tutorials/GAMM.html

We aimed to model the differences in HR between the two species over time. We decided to use an ordered factor for the species which will allow us to detect significant differences between the two groups over time.

Data file

The data file is available in the Dyrad data repositrory. DOI:https://doi.org/10.5061/dryad.6tt0h5s

Variables

Object_ID = the unique ID of the bears
HeartRate = daily mean heart rate in bpm
GMT_date = date for the mean heart rate
DenningDay = first day of the year (yday) that the bears daily mean HR was <40 bpm, assumed start of hibernation
BodyMass = weight of the bear in Kg
PreDenDay = the time difference of the GMT_date to the DenningDay in days
Species = Bear species

Packages

```r
library(dplyr)  ## 0.7.6
library(itsadug) ## 2.3
library(lubridate) ## 1.7.4
library(mgcv) ## 1.8-24
library(tidyverse) ## 1.2.1
```

Prepare the data

Create factors and dates.

```r
hr$Object_ID <- as.factor(hr$Object_ID)
hr$yday <- yday(ymd_hms(hr$GMT_date,tz="UTC"))
```

Create an ordered group factor for the species.

```r
hr$oSpecies <- as.ordered(hr$Species)
contrasts(hr$oSpecies) <- "contr.treatment"
```

Specifying the start of each time series. i.e. first day of data for each bear.

```r
hr <- start_event(hr,"PreDenDay","Object_ID")
```

Look at the data

First the daily mean HR against the day of the year (yday).

```r
hr %>%
  ggplot(aes(yday,HeartRate,color=Species))+
  geom_point()+
  scale_color_manual(values=c("black","brown"))
```

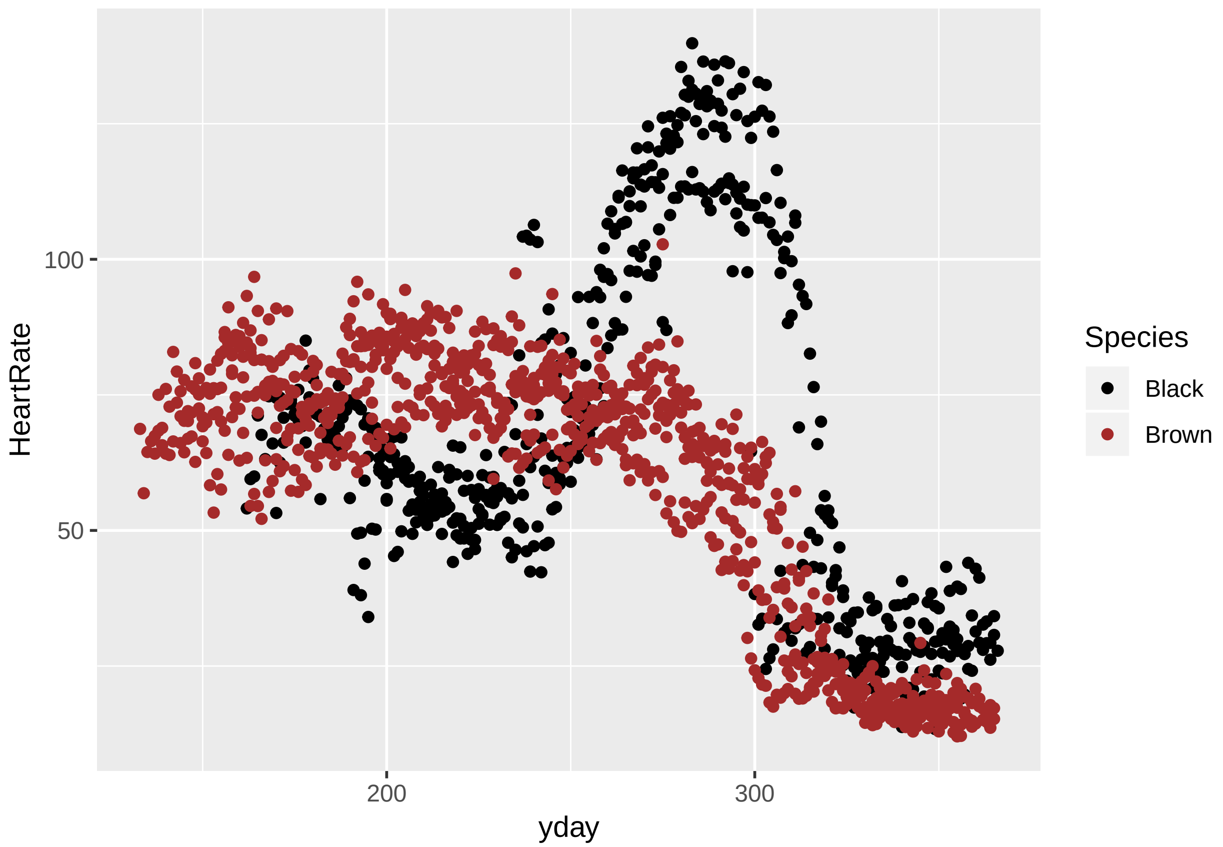

Now the data zeroed on the day of start of hibernation.

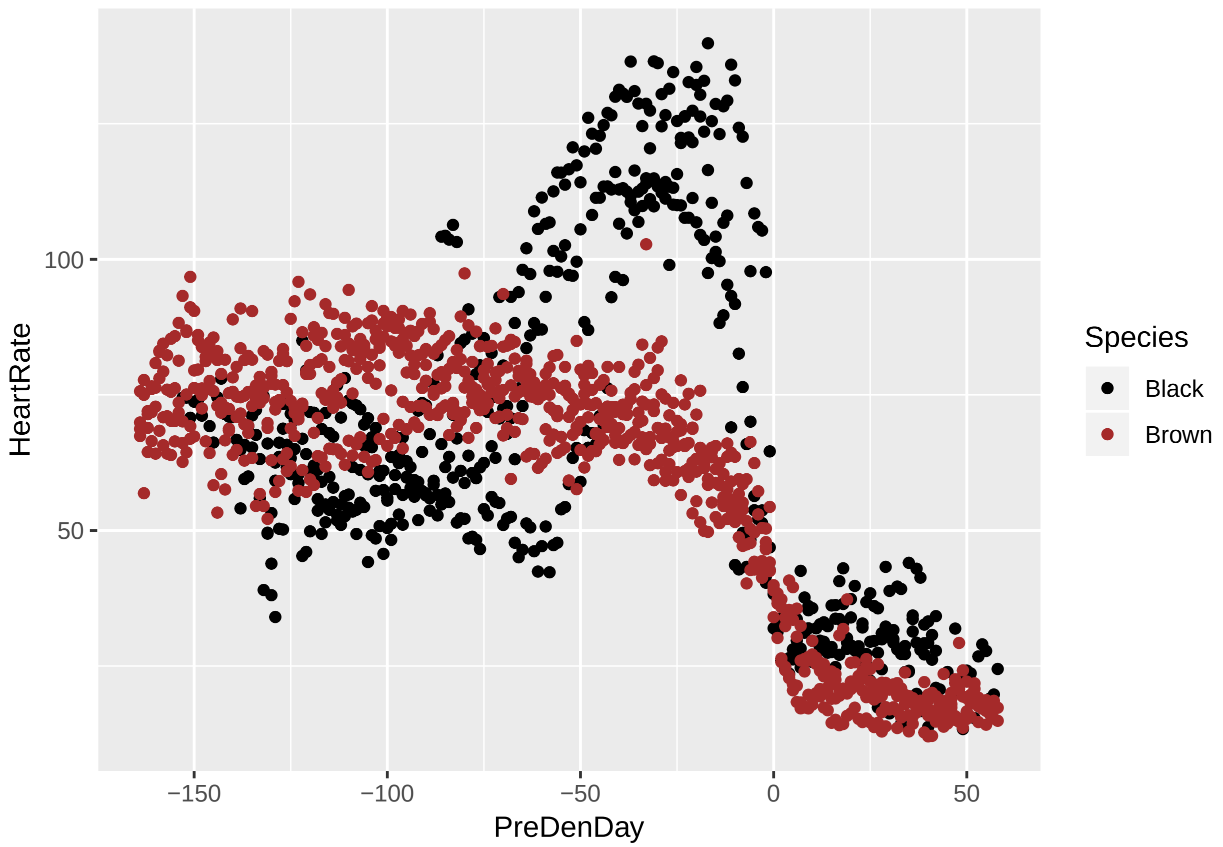

Model selection

We used gamm models and the function “bam”. We decided a priori on the variables. We used a smoother on the days prior to hibernation as the time variable because we expected non-linearity. We need the species as the grouping factor and the ID as a random component. We decided to create an interaction like term to model different trends over time for each species represented in the model as s(PreDenDay, by=oSpecies). First we compare models with increasing random complexity and select based on fREML and AIC. We used default k for all smoother parameters.

Random intercept per ID:

```
m1 <- bam(HeartRate ~
            oSpecies +
            s(PreDenDay) +
            s(PreDenDay, by=oSpecies) +
            s(Object_ID, bs="re"),
            data=hr)
summary(m1)
```

```
##
## Family: gaussian
## Link function: identity
##
## Formula:
## HeartRate ~ oSpecies + s(PreDenDay) + s(PreDenDay, by = oSpecies) +
##     s(Object_ID, bs = "re")
```

```
##
## Parametric coefficients:
##             Estimate Std. Error t value Pr(>|t|)
## (Intercept)   62.739      1.691  37.094   <2e-16 ***
## oSpecies.L    -5.100      2.392  -2.132   0.0332 *
## ---
## Signif. codes:  0 '***' 0.001 '**' 0.01 '*' 0.05 '.' 0.1 ' ' 1
##
## Approximate significance of smooth terms:
##                                edf Ref.df     F p-value
## s(PreDenDay)                 8.883  8.986 508.4  <2e-16 ***
## s(PreDenDay):oSpeciesBrown 8.691  8.962 180.8  <2e-16 ***
## s(Object_ID)                 4.869  5.000  37.5  <2e-16 ***
## ---
## Signif. codes:  0 '***' 0.001 '**' 0.01 '*' 0.05 '.' 0.1 ' ' 1
##
## R-sq.(adj) =  0.871   Deviance explained = 87.3%
## fREML = 5471.6  Scale est. = 103.1     n = 1450
```

Random intercept and slope per ID.

```
m2 <- bam(HeartRate ~
            oSpecies +
            s(PreDenDay) +
            s(PreDenDay, by=oSpecies) +
            s(Object_ID, bs="re")+
            s(Object_ID, PreDenDay, bs="re"),
            data=hr)
summary(m2)
```

```
##
## Family: gaussian
## Link function: identity
##
## Formula:
## HeartRate ~ oSpecies + s(PreDenDay) + s(PreDenDay, by = oSpecies) +
##     s(Object_ID, bs = "re") + s(Object_ID, PreDenDay, bs = "re")
##
## Parametric coefficients:
##             Estimate Std. Error t value Pr(>|t|)
## (Intercept)   62.648      1.480  42.342   <2e-16 ***
## oSpecies.L    -5.030      2.092  -2.404   0.0164 *
## ---
## Signif. codes:  0 '***' 0.001 '**' 0.01 '*' 0.05 '.' 0.1 ' ' 1
##
## Approximate significance of smooth terms:
##                                edf Ref.df     F  p-value
## s(PreDenDay)                 8.887  8.987 499.5  < 2e-16 ***
## s(PreDenDay):oSpeciesBrown 8.697  8.963 135.0  < 2e-16 ***
## s(Object_ID)                 4.620  5.000 108.3 9.54e-07 ***
## s(Object_ID,PreDenDay)       4.607  5.000 233.4  < 2e-16 ***
## ---
## Signif. codes:  0 '***' 0.001 '**' 0.01 '*' 0.05 '.' 0.1 ' ' 1
##
## R-sq.(adj) =  0.877   Deviance explained = 87.9%
```

```
## fREML = 5442.5  Scale est. = 98.293    n = 1450
```

```r
compareML(m1,m2)
```

```
## m1: HeartRate ~ oSpecies + s(PreDenDay) + s(PreDenDay, by = oSpecies) +
##     s(Object_ID, bs = "re")
##
## m2: HeartRate ~ oSpecies + s(PreDenDay) + s(PreDenDay, by = oSpecies) +
##     s(Object_ID, bs = "re") + s(Object_ID, PreDenDay, bs = "re")
##
## Chi-square test of fREML scores
## -----
##   Model    Score Edf Difference    Df   p.value Sig.
## 1    m1 5471.621   7
## 2    m2 5442.471   8     29.150 1.000 2.250e-14  ***
##
## AIC difference: 64.51, model m2 has lower AIC.
```

Model 2 has lower AIC and fREML score. Some diagnostics are needed.

Residual auto correlation is not ideal

```r
acf_resid(m2,split_pred="Object_ID",main="m2")
```

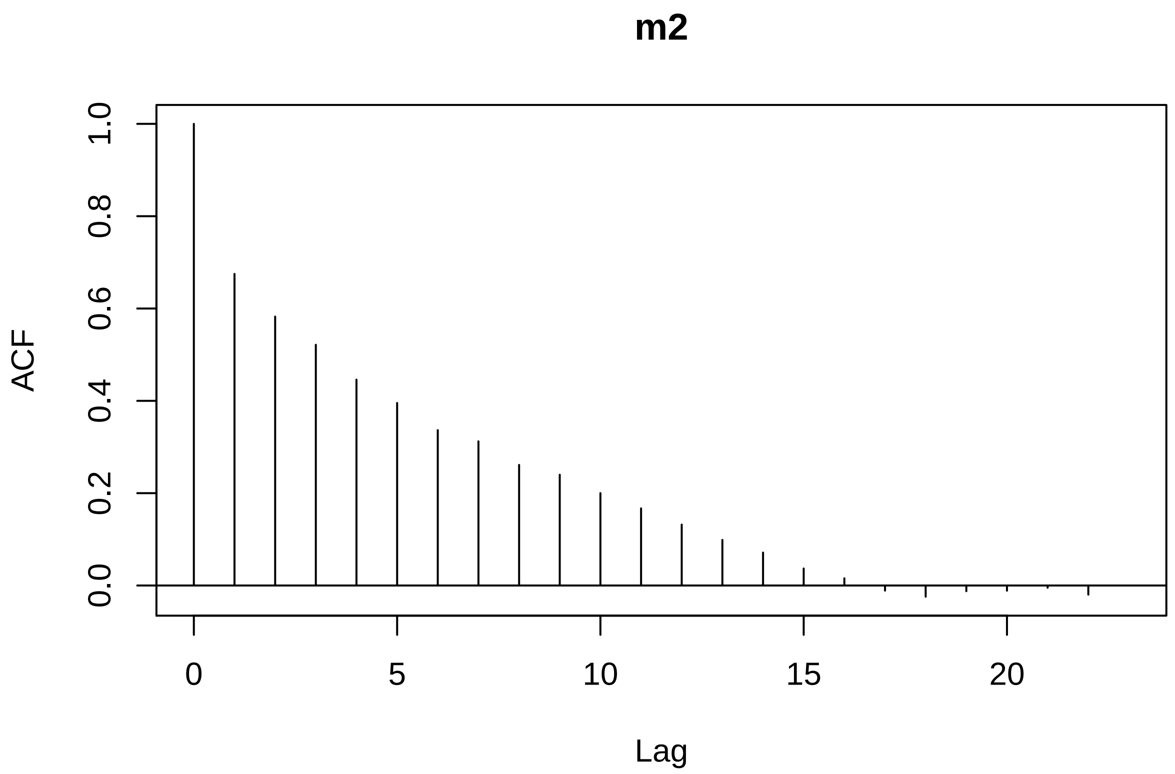

We can implement an auto regression function to decrease the problem. As starting point for the AR starting value we use the correlation factor of the residuals at the first lag.

```r
acf(resid(m2), plot=FALSE)$acf[2]
```

```
## [1] 0.8269472
```

```r
m3 <- bam(HeartRate ~
            oSpecies +
            s(PreDenDay) +
            s(PreDenDay, by=oSpecies) +
            s(Object_ID, bs="re")+
            s(Object_ID, PreDenDay, bs="re"),
            AR.start=hr$start.event, rho=0.8,
            data=hr,method="ML")
summary(m3)
```

```
##
## Family: gaussian
## Link function: identity
##
## Formula:
## HeartRate ~ oSpecies + s(PreDenDay) + s(PreDenDay, by = oSpecies) +
##     s(Object_ID, bs = "re") + s(Object_ID, PreDenDay, bs = "re")
##
## Parametric coefficients:
##             Estimate Std. Error t value Pr(>|t|)
## (Intercept)   62.614      1.316   47.57  < 2e-16 ***
## oSpecies.L    -4.991      1.862   -2.68  0.00745 **
## ---
## Signif. codes:  0 '***' 0.001 '**' 0.01 '*' 0.05 '.' 0.1 ' ' 1
##
## Approximate significance of smooth terms:
##                              edf Ref.df      F p-value
## s(PreDenDay)               8.488  8.830 71.493 < 2e-16 ***
## s(PreDenDay):oSpeciesBrown 7.378  8.296 21.868 < 2e-16 ***
## s(Object_ID)               2.383  5.000  2.002 0.03817 *
## s(Object_ID,PreDenDay)     3.088  5.000  3.473 0.00418 **
## ---
## Signif. codes:  0 '***' 0.001 '**' 0.01 '*' 0.05 '.' 0.1 ' ' 1
##
## R-sq.(adj) =  0.872   Deviance explained = 87.4%
## -ML = 4588.6  Scale est. = 85.911    n = 1450
```

To fit m3 the method="ML" is added to get more apropriate predictions.

Using itsadug::check_resid() we can investigate residual correlation with and without AR1 structure. In the plot "ACF resid(m3)" residual ACF with AR1 structure is indicated by solid black bars, residual ACF without AR1 structure is indicated by grey bars. In this case - much better.

Residual distribution is not ideal but probably as good as it can be with n=7.

```r
par(mfrow=c(2,2))
check_resid(m3,split_pred = "Object_ID", ask=F)
```

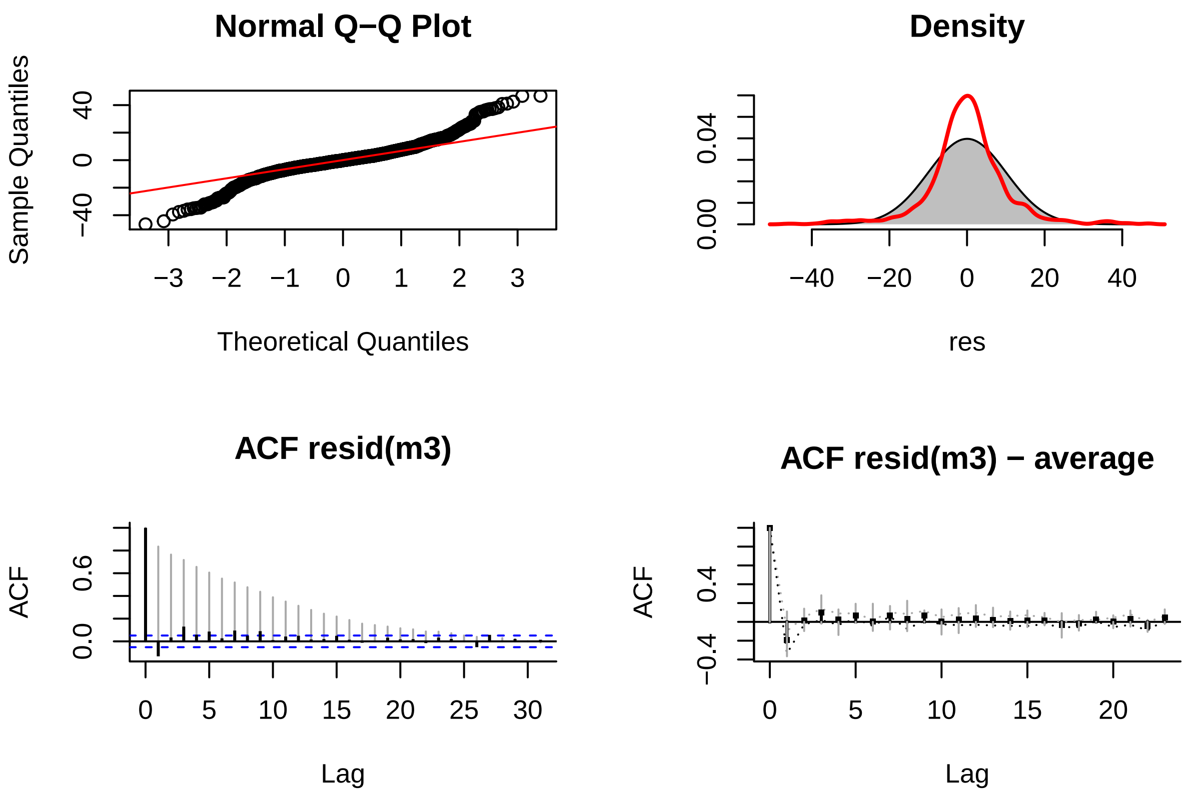

So far we used default k for the smooth terms, we can check:

```
gam.check(m3)
```

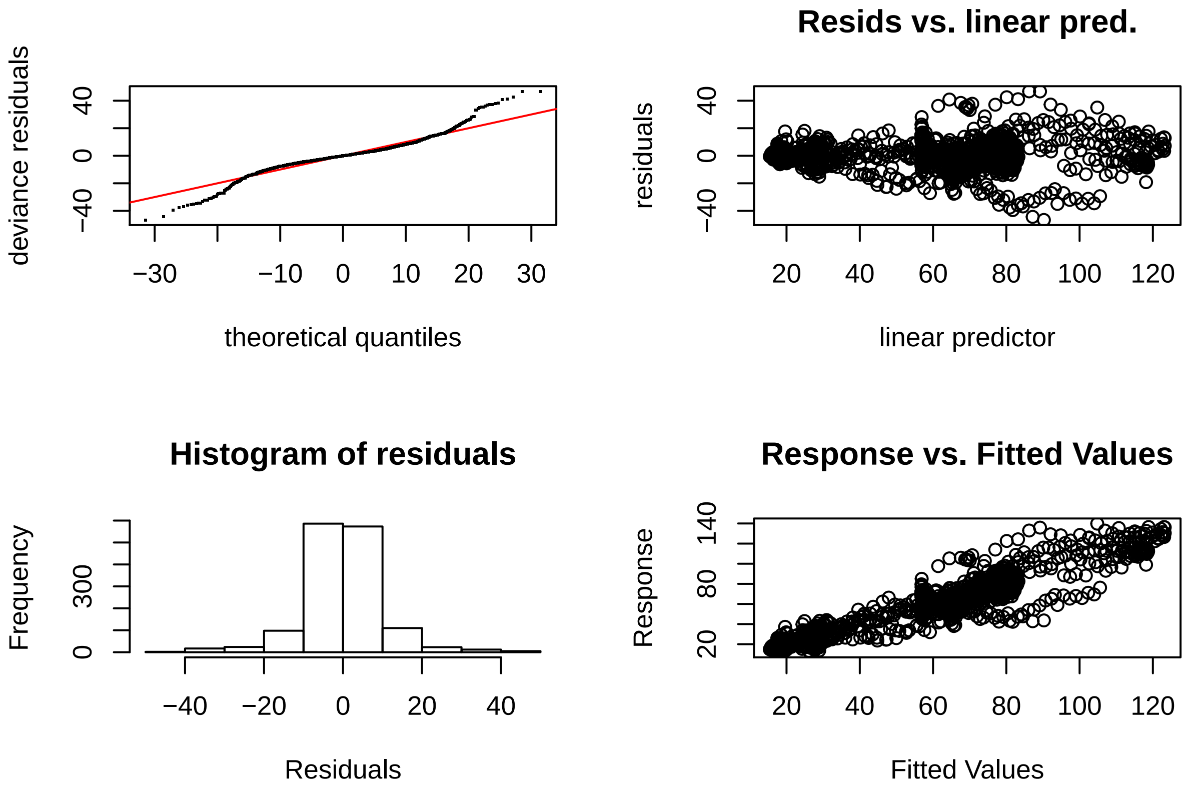


```
##
## Method: ML   Optimizer: outer newton
## full convergence after 5 iterations.
## Gradient range [-0.001800145,3.771811e-06]
## (score 4588.631 & scale 85.91094).
## Hessian positive definite, eigenvalue range [0.6545839,725.0458].
## Model rank =  34 / 34
##
## Basis dimension (k) checking results. Low p-value (k-index<1) may
## indicate that k is too low, especially if edf is close to k'.
##
##                                k'  edf k-index p-value
## s(PreDenDay)                 9.00 8.49    1.29       1
## s(PreDenDay):oSpeciesBrown 9.00 7.38    1.29       1
## s(Object_ID)                 7.00 2.38      NA      NA
## s(Object_ID,PreDenDay)       7.00 3.09      NA      NA
```

Again residual distribution is not ideal but at least it is symmetric around 0. K indicates how much "non-linearity" we allow each smoother. In this case we "over smooth" but in the output it reflects the data much better as if we would use k=5. Probably because the brown bears are much closer to linear than the black bears. Using lower k will lead to a significant difference for most of the time which is obviously wrong. Using k=10 or k=20 the output does not differ substantially. We keep default k=10.

We keep m3 and plot the difference between black and brown bears. We use simultaneous calculation of the confidence intervals (ci) instead of the default pointwhise. This will calculate the ci for the entire smooth instead for each point of time, involving more (accurate) uncertainty. It is based on simulations and for reproducability we use set.seed(42) ensuring the simulations to start at the same point.

```r
par(mfrow=c(1,1))
set.seed(42)
p<- plot_diff(m3, view="PreDenDay",comp=list(oSpecies=c("Black","Brown")),rm.ranef = T,sim.ci=T)
```

```
## Summary:
##  * PreDenDay : numeric predictor; with 200 values ranging from -164.000000 to 58.000000.
##  * Object_ID : factor; set to the value(s): W1304. (Might be canceled as random effect, check below.)
##  * NOTE : The following random effects columns are canceled: s(Object_ID),s(Object_ID,PreDenDay)
##
##  * Simultaneous 95%-CI used :
##      Critical value: 2.945
##      Proportion posterior simulations in pointwise CI: 1 (10000 samples)
##      Proportion posterior simulations in simultaneous CI: 1 (10000 samples)
##
```

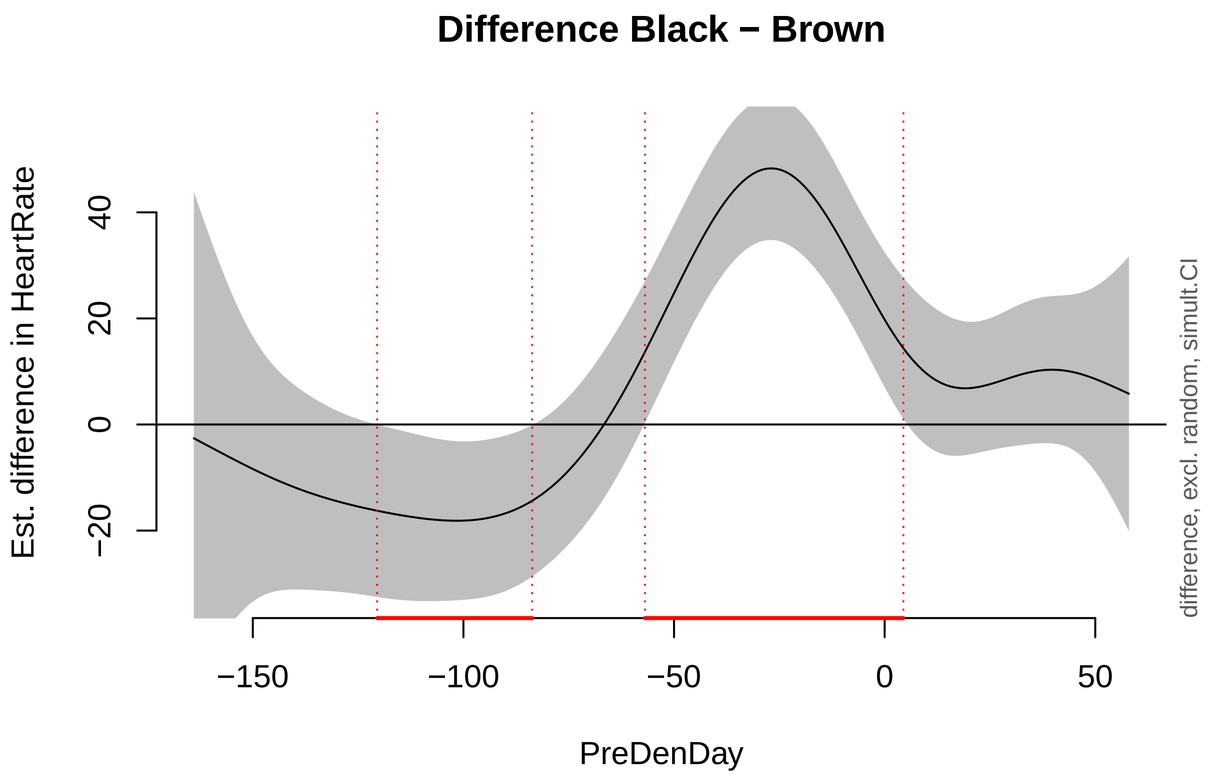


```
##
## PreDenDay window(s) of significant difference(s):
##  -120.492462 - -83.678392
##  -56.904523 - 4.452261
```

To get fitted and predicted data

```r
d <- get_difference(m3,comp=list(oSpecies=c("Black","Brown")),
                    cond=list(PreDenDay=(seq(min(hr$PreDenDay),
                    max(hr$PreDenDay),by=1))),rm.ranef = T, sim.ci = T)
```

```
## Summary:
##  * PreDenDay : numeric predictor; with 223 values ranging from -164.000000 to 58.000000.
```

```
##  * Object_ID : factor; set to the value(s): W1304. (Might be canceled as random effect, check below.)
##  * NOTE : The following random effects columns are canceled: s(Object_ID),s(Object_ID,PreDenDay)
##
##  * Simultaneous 95%-CI used :
##      Critical value: 2.965
##      Proportion posterior simulations in pointwise CI: 1 (10000 samples)
##      Proportion posterior simulations in simultaneous CI: 1 (10000 samples)
##
```

R version 3.5.1 (2018-07-02) Platform: x86_64-w64-mingw32/x64 (64-bit) Running under: Windows >= 8 x64 (build 9200)
